# Supplementary material for: Inactivating mutations and X-ray crystal structure of the tumor suppressor OPCML reveal cancer-associated functions
Source: Nat Commun. 2019 Jul 17;10:3134. doi: 10.1038/s41467-019-10966-8 (PMC6637204; doi:10.1038/s41467-019-10966-8)
Supplement: Supplementary file 1 — Supplementary Information [file 41467_2019_10966_MOESM1_ESM.pdf]

Supplementary information

Inactivating mutations and crystal structure of the tumor suppressor OPCML reveal new cancer-associated functions

JR Birtley, M Alomary et al.

A)

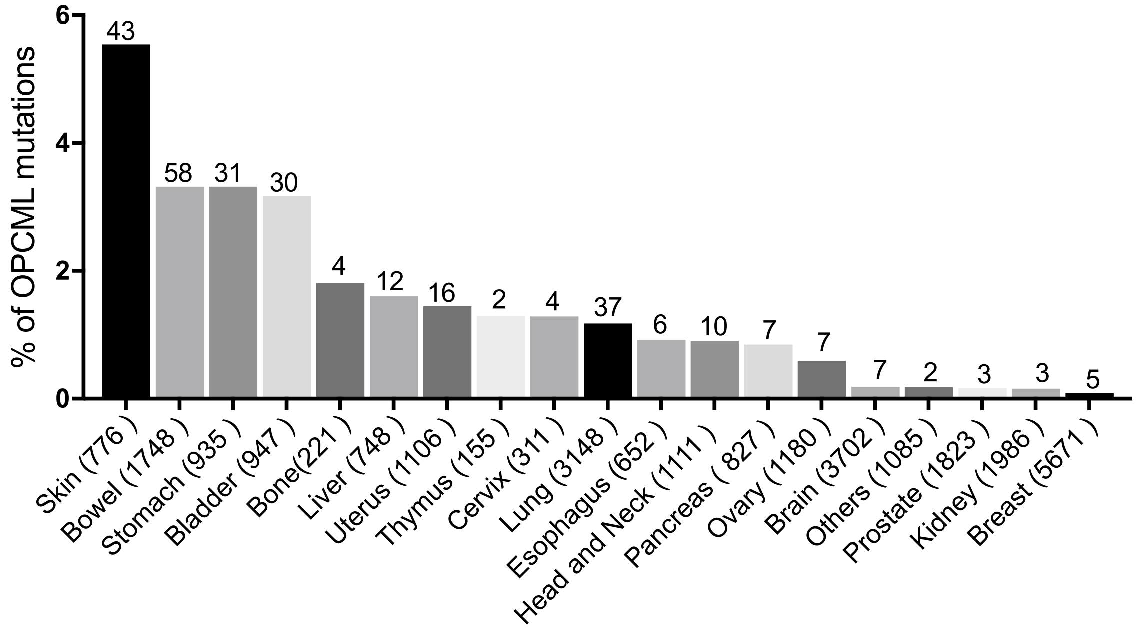

B)

	N-term	D1	D2	D3	C-term	Total
**Residue numbers**	1-41	42-134	135-222	223-314	315-345	345
**Number of residues**	41	93	88	92	31	345
**% of OPCML**	11.9	**26.9**	25.5	26.7	9.0	100
**Total number of mutations**	23	111	67	65	21	287
**Total % of mutations**	8.0	**38.7**	23.3	22.6	7.3	100
**Actual positions mutated**	11	40	24	34	9	118
**% of positions mutated**	9.3	**33.9**	20.3	28.8	7.6	100

Supplementary Figure 1. OPCML is mutated in different tumor types. (A) The graph shows the percentage of patients who present mutations in the OPCML gene (y-axis) classified per cancer type. The numbers in brackets on the x-axis indicate the total number of patients for each cancer type, while the numbers above the bars show the actual number of patients who have mutations. **(B)** The table indicates the actual number and the relative percentage of the residues present in OPCML, the mutations identified in the databases and the amino acids mutated in the different domains of OPCML.

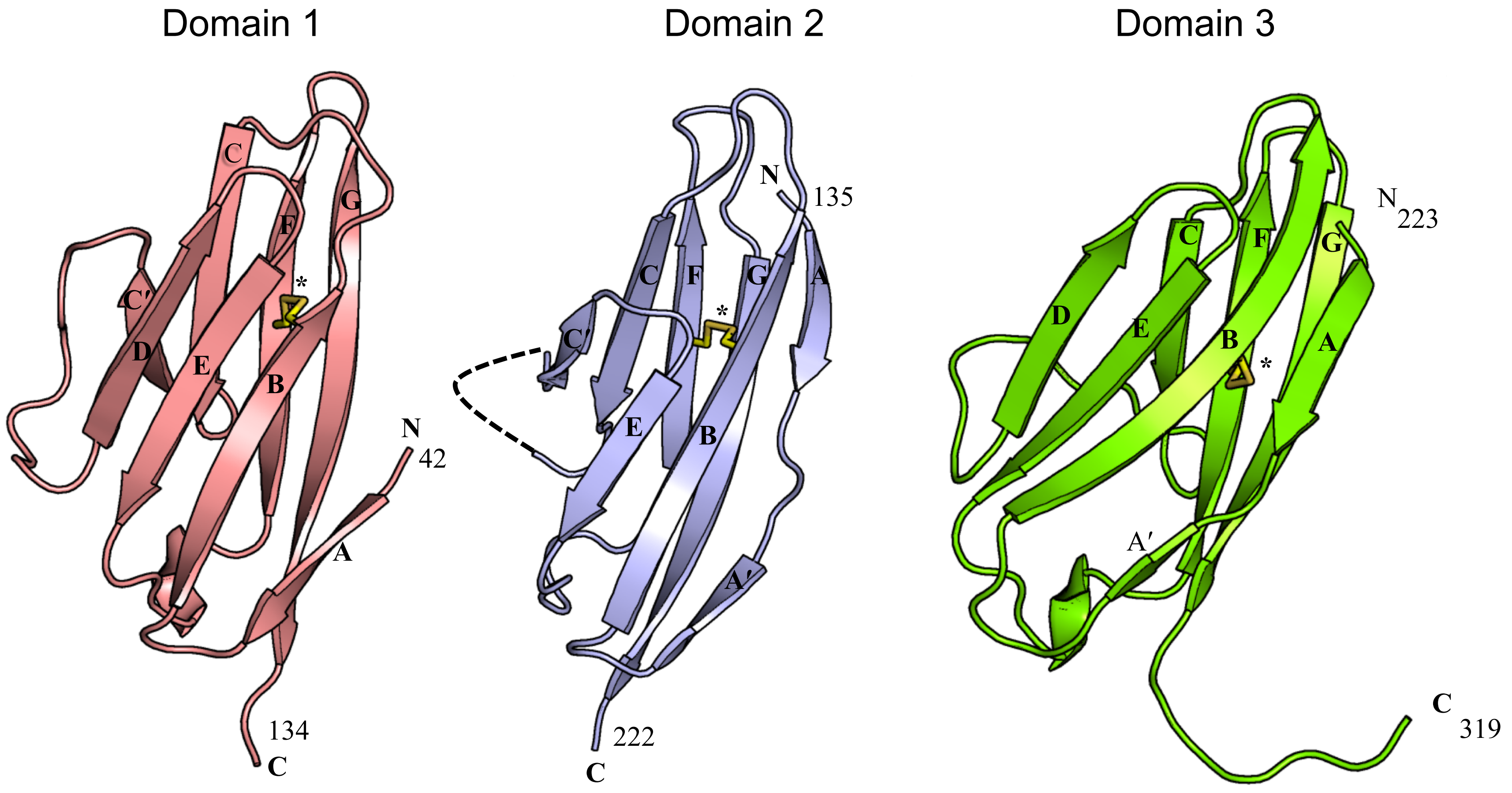

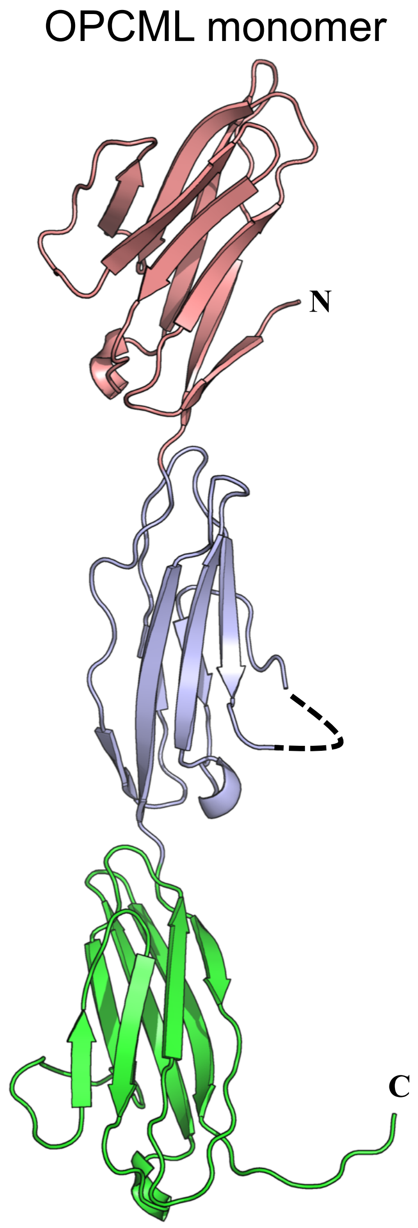

Supplementary Figure 2. Topology diagrams of domains 1, 2 and 3 from OPCML. Domain 1, shown in salmon, belongs to the V-set subfamily of Ig domains and consists of nine β-strands. The AGFCC′ forms one sheet and BED the other. Strand A is displaced somewhat because the C-terminal end from a neighboring molecule (residues 315-9) inserts between the B and G strands to make crystal contacts. Domain 2 (blue) is an I2-set Ig domain; strand D is absent. The loop between the C and C' strands (residues 175-179) is missing due to disorder in the crystals and is shown as a dashed black line. Domain 3 (green) exhibits an I1-set topology25 with two β-sheets, ABED and A′GFC. The A–A′ segmented β-strand, crosses over between B and G respectively. Disulfide bridges connect all B and F strands and are indicated (*). The N- and C- termini are indicated and are numbered. The right panel shows a monomer of OPCML, with the same color scheme used on the left 3 panels.

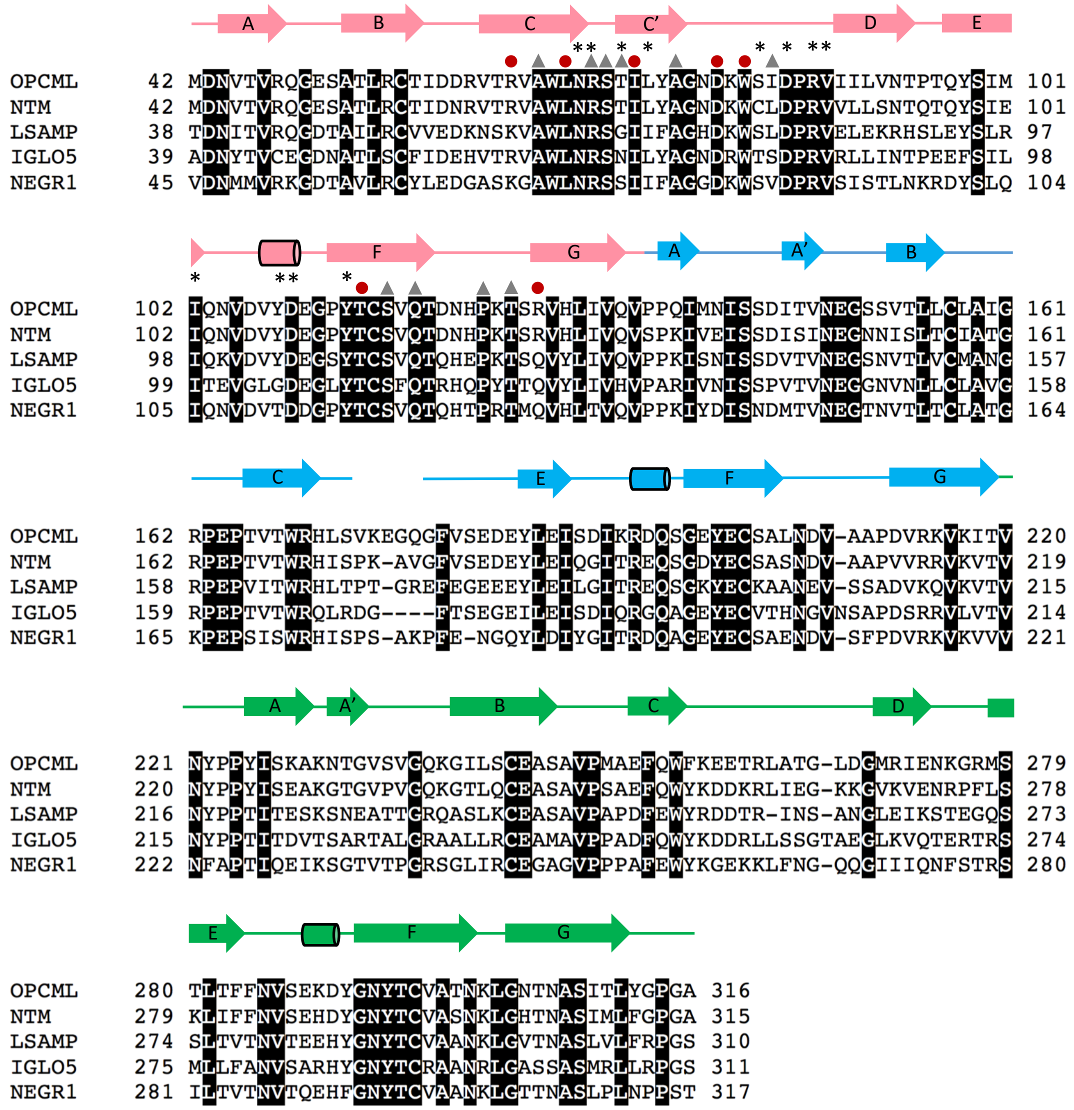

Supplementary Figure 3. Structure-based multiple sequence alignment of the IgLON family. Residues conserved across the family are highlighted in black with white lettering. OPCML domain 1 (pink), domain 2 (blue) and domain 3 (green) are shown above the alignment with beta strands as arrows and alpha helices as cylinders. The residues involved in maintaining stacking interaction at the D1-D1 interface are highlighted with red spheres and include Arg 65, Leu 69, Ile 74, Asp 80, Trp 82, Thr 114 and Arg 127 (shown structurally on Figure 1C). The side chains of other residues making contacts at the D1-D1 interface are highlighted with gray triangles and include Ala 67, Arg 71, Ser 72, Thr 73, Ala 77, Ile 84 Ser 116, Gln 118, Pro 123 and Thr 125. Asn 70, residues that make contacts with Asn 70 or to sugar residues attached to Asn 70 are shown with an asterisk (Arg 71, Thr 73, Leu 75, Ser 83, Asp 85, Arg 87, Val 88, Ile 102, Tyr 108, Asp 109 and Tyr 113). These are shown structurally in Supplemental Fig 4. The N-terminal signal sequence and the C-terminal GPI anchor site are not shown for simplicity. Secondary structure for OPCML residues 174-178 are not shown as they are missing in the electron density. The UNIPROT accession numbers for the amino acids used above are given in parentheses followed by percentage sequence identity with OPCML: OPCML (Q14982), NTM (Q9P121, 76%), LSAMP (Q13449, 56%), IGLO5 (A6NGN9, 50%) and NEGR1 (Q7Z3B1, 48%).

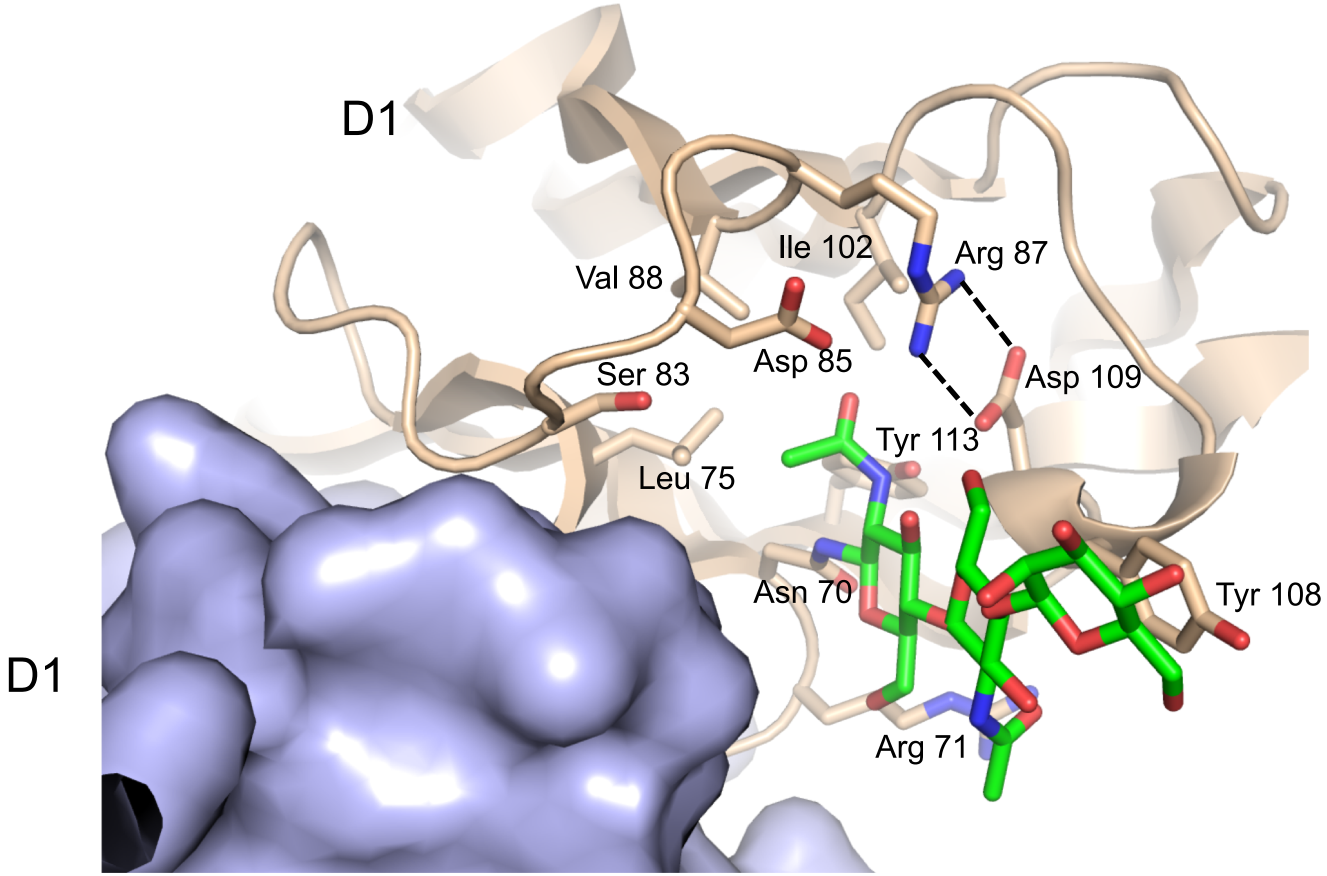

Supplementary Figure 4. Amino acid residues at the D1-D1 interface. Amino acid residues that make contact with Asn 70 and the NAG-NAG-MAN attached to Asn 70 are shown. D1 from one homodimer is shown in blue space fill and the other D1 as cartoon representation. The NAG-NAG-MAN (shown in green) is covalently attached to Asn 70 and is in stick representation. Other residues involved in contacting Asn 70 and to the sugar residues and shown in stick representation and include Arg 71, Leu 75, Ser 83, Asp 85, Arg 87, Val 88, Ile 102, Tyr 108, Asp 109 and Tyr 113. Thr 73 is out of view. A salt bridge interaction between Arg 87 and Asp 109 are shown by dashed black lines.

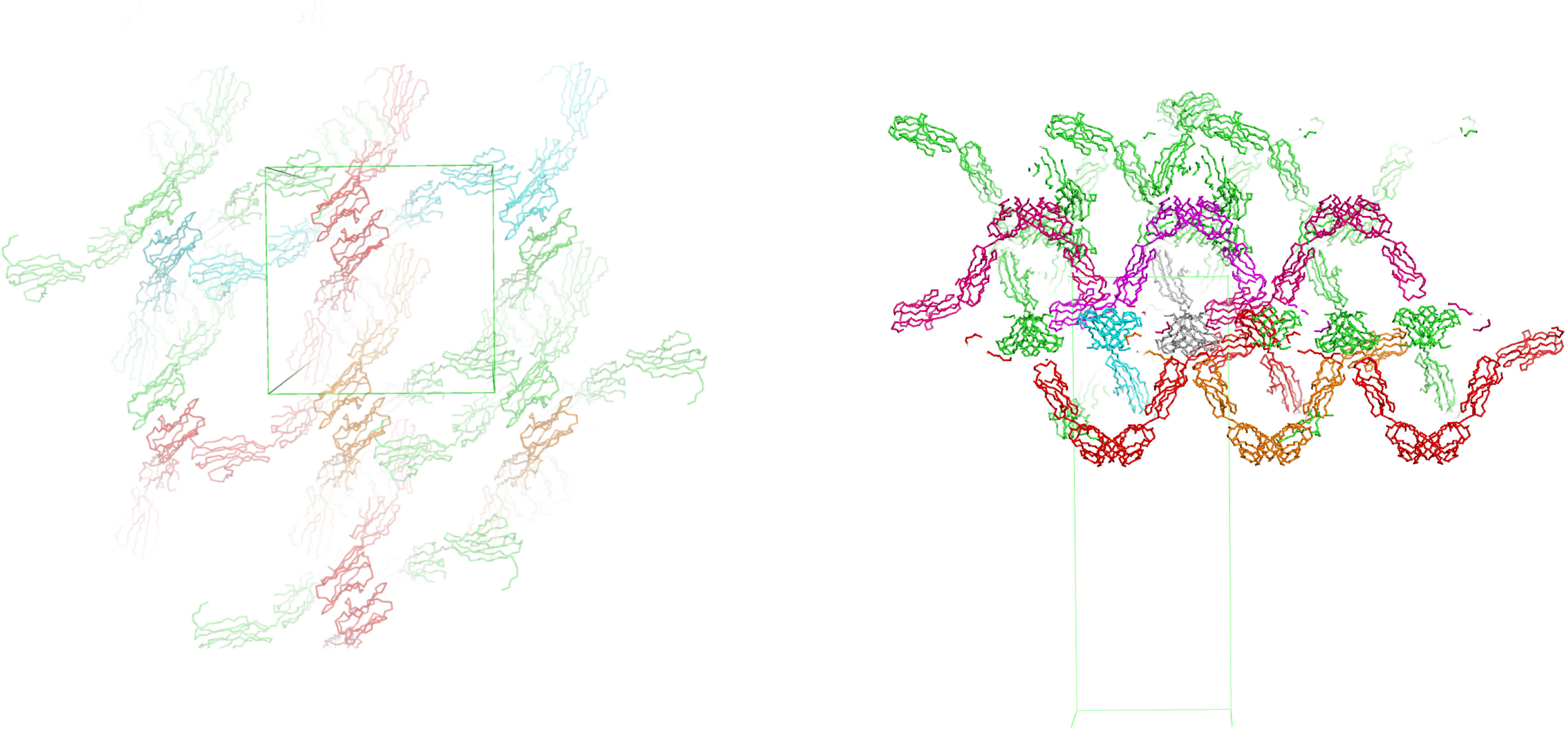

Supplementary Figure 5. Packing of OPCML in the crystal lattice. Orthogonal views of OPCML packing interactions as seen in the crystal lattice. The unit cell is shown in both and the OPCML peptide backbone shown as a trace.

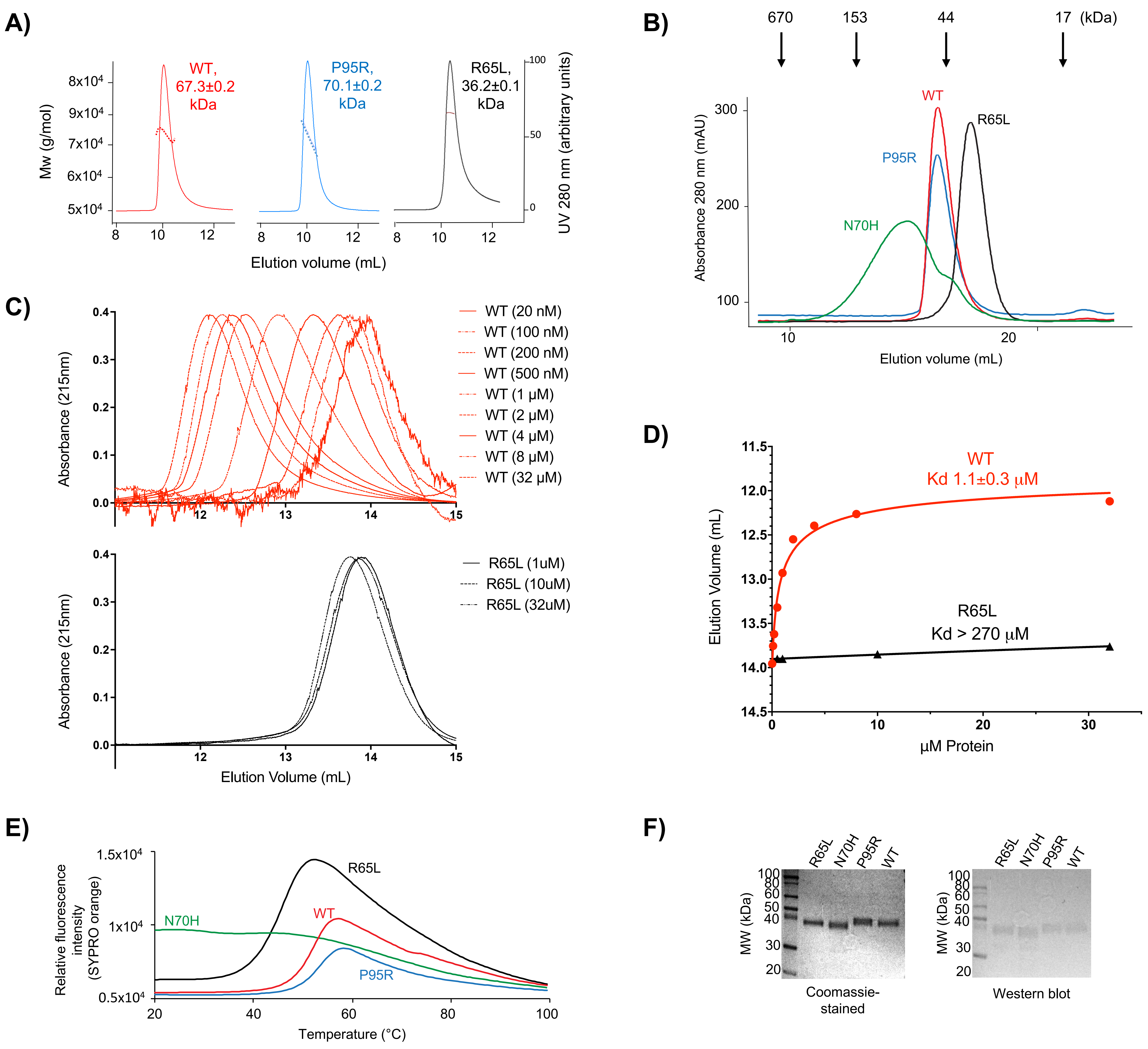

Supplementary Figure 6. Biophysical characterization of WT and point-mutated OPCML. WT and P95R are predominantly dimeric in solution and R65L is predominately monomeric. **(A)** Gel filtration analysis of WT and OPCML mutants. The elution profiles of WT (red), P95R (blue), R65L (black) and N70H (green) is shown along with molecular weight size standards (in kDa). WT and P95R elute similarly, whilst R65L elutes more slowly. N70H is aggregated. **(B)** The molecular masses of WT, P95R, R65L and N70H (shown in red, blue and black, respectively) were calculated from the elution profile given by SEC-MALS. The calculated masses for WT was 67.3 ±0.2 kDa, P95R 70.1 ±0.2 kDa and R65L 36.2 ±0.1 kDa. OPCML has a molecular mass of approximately 31.7 kDa, as calculated from the primary sequence. **(C)** Concentration dependence of gel filtration profiles for WT (20 nM to 32 µM) and R65L (1 to 32 µM). **(D)** Estimation of monomer-dimer dissociation constant Kd. Peak positions from panel C (circles for WT and triangles for R65L) were fit to an equation describing the monomer-dimer equilibrium (see Methods) showing fit values for WT (95% confidence interval 0.565 to 1.997 µM) and R65L (840 ± 630 µM, 95% confidence interval lower limit 270 µM with no upper limit determined). **(E)** Melting curve profiles for WT, P95R, R65L and N70H (shown in red, blue, black and green respectively) were generated by a SYPRO orange-based thermofluor thermal shift assay. Purified recombinant proteins were heated from room temperature to 95°C, in 0.5°C increments. WT melted at an average of 55.5 °C and P95R 57.0 °C, indicating they have comparable thermal stability. R65L melted at an average of 51.5 °C. All measurements were performed in triplicate. **(F)** SDS PAGE and western blot analysis of purified R65L, N70H, P95R and WT. 0.5 µg of each protein was loaded per lane.

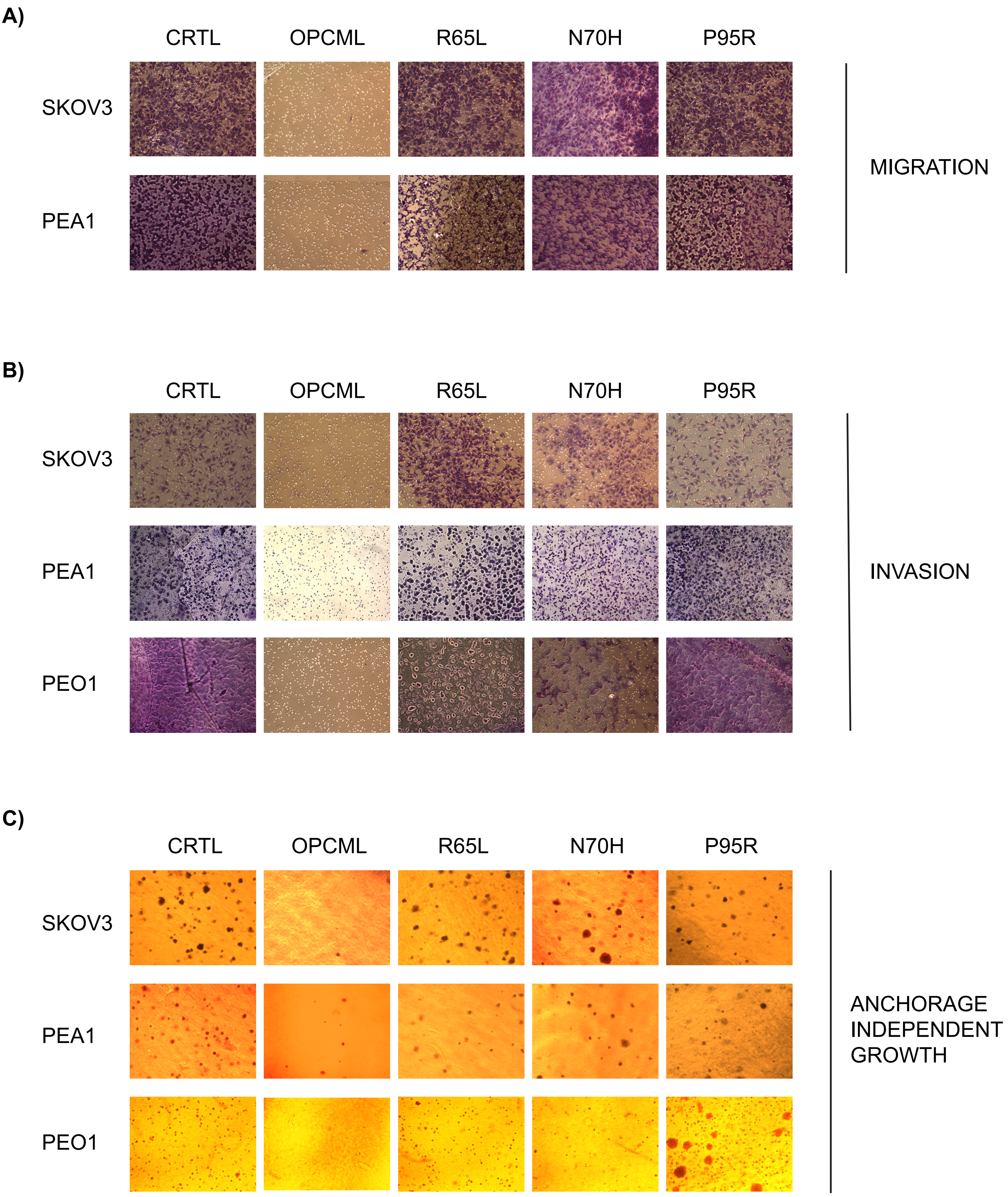

Supplementary Figure 7. OPCML mutants are impaired in migration, invasion and anchorage-independent growth. SKOV3, PEA1 and PEO1 ovarian cell lines were transduced with an empty vector (CTRL), wild-type (OPCML) or mutant (R65L, N70H and P95R) OPCML and tested for migration **(A)** and invasion **(B)** in transwell assays Representative images of cells that have migrated or invaded, stained with crystal violet. Colonies formed in in agarose following anchorage-independent growth are stained with Red **(C)**.

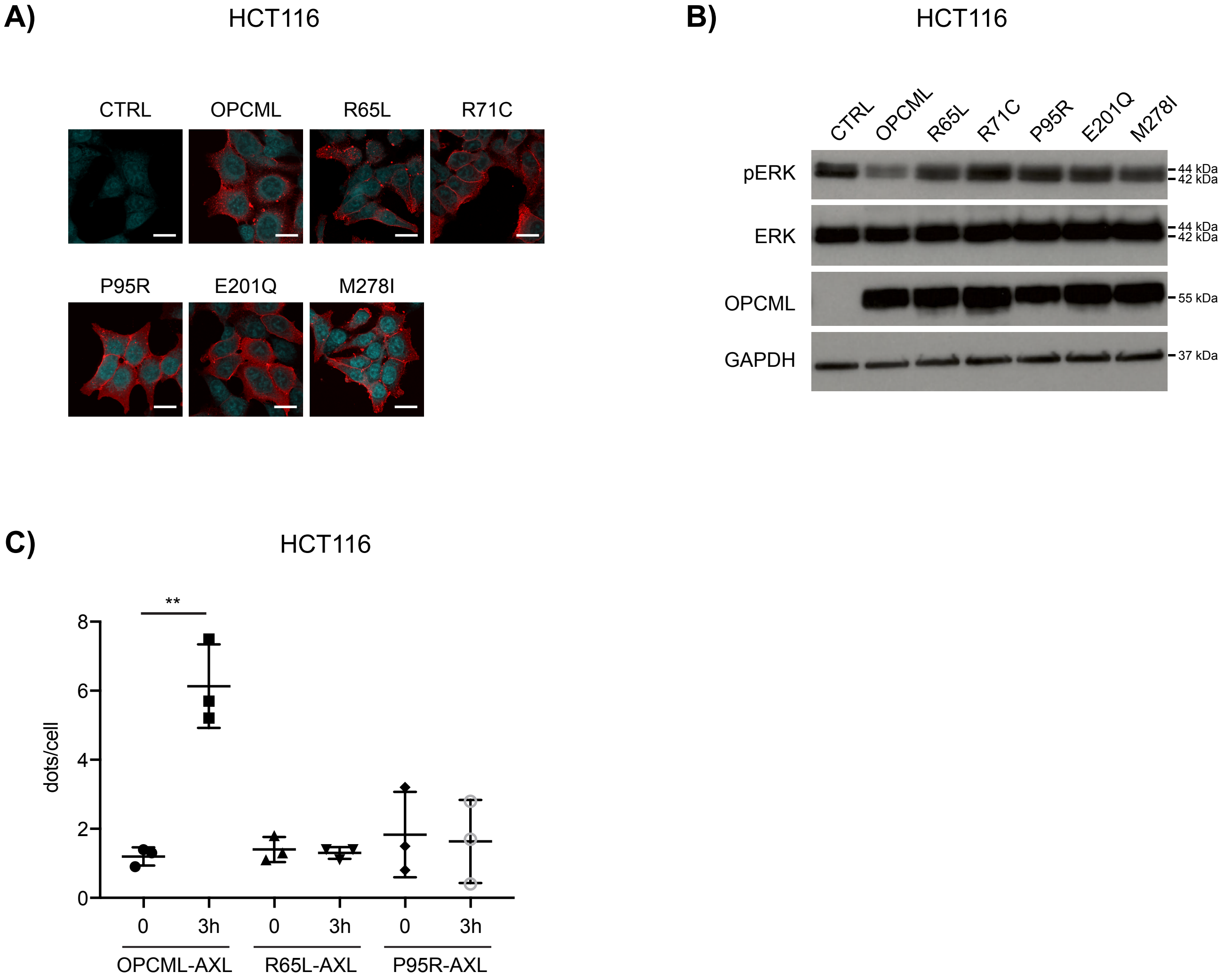

Supplementary Figure 8. OPCML mutants show impaired tumour suppressor functions in HCT116 cells. (A) Colorectal HCT116 cells were stably transduced with the indicated constructs and protein expression was verified by immunofluorescence with an anti-OPCML antibody (red). Cell nuclei were stained with DAPI (cyan). Scale bar = 20μm. **(B)** Cells were grown in full medium conditions and their signaling analyzed by western blotting with the indicated antibodies. **(C)** Cells were starved and then stimulated for 3h with Gas6, the interaction of OPCML WT, R65L and P95R with AXL was measured by DuoLink. The graph shows the mean ± s.e.m of 3 independent experiments. Student t-test ** $p<0.01$.

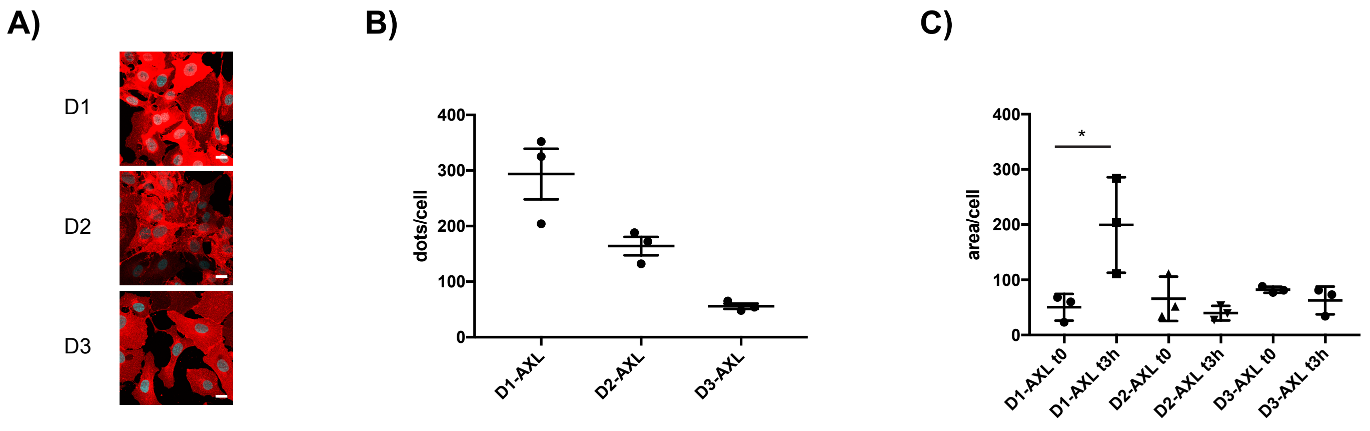

Supplementary Figure 9. OPCML interacts with AXL mainly via the D1 domain. (A) SKOV3 cells were stably transduced with D1, D2 or D3, which are all HA-tagged, and stained in red with an anti-HA antibody. Nuclei are stained by DAPI (cyan). Scale bar = 20μm. **(B)** Cells were grown in full medium and the interaction between the different domains of OPCML and AXL was measured by DuoLink. The number of dots was quantified and graph shows the mean ± s.e.m of 3 independent experiments. **(C)** Cells were starved and then stimulated with Gas6 for 3h. The interaction between OPCML's domains and AXL was measured by DuoLink. The total area covered by the signal was quantified and the graph shows the mean ± s.e.m of 3 independent experiments. Student t-test: * $p<0.05$.

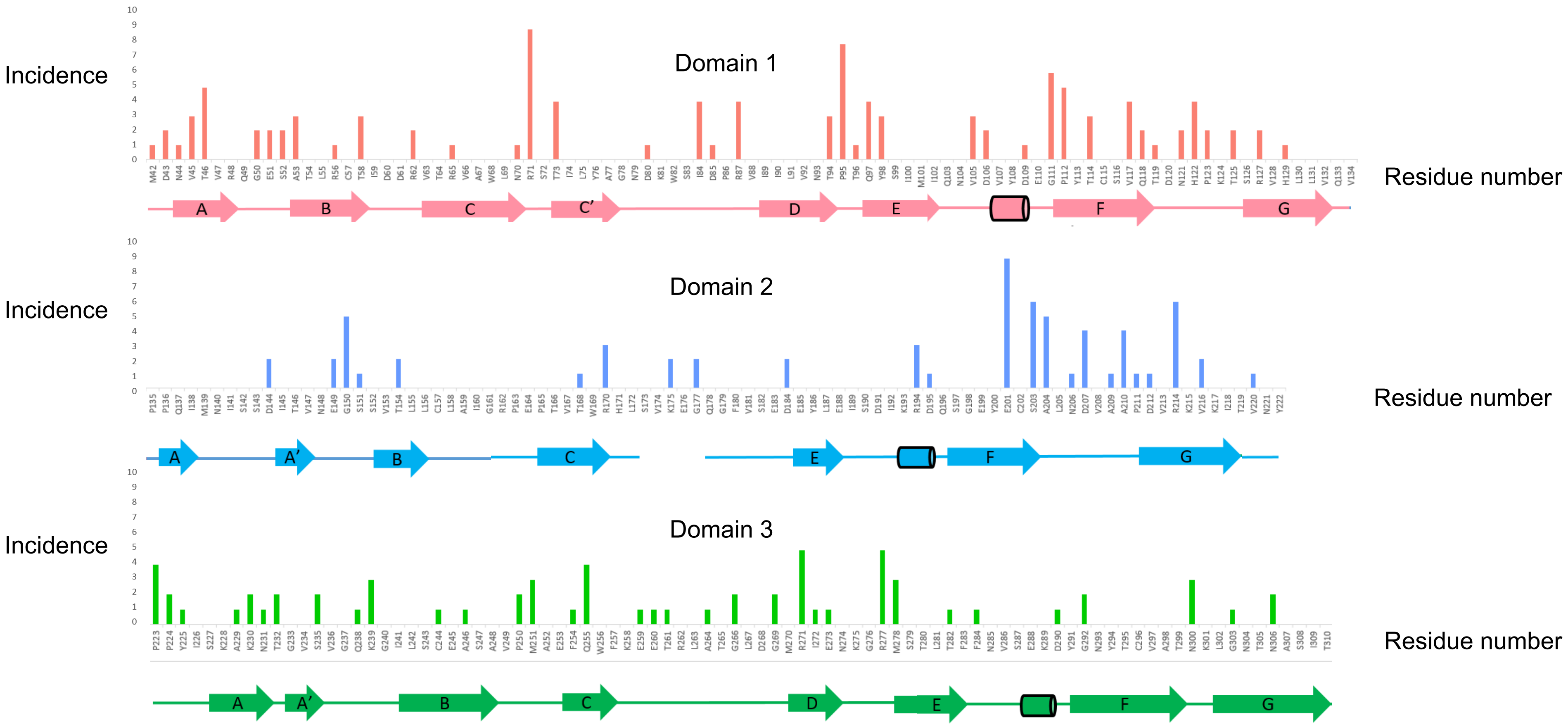

Supplementary Figure 10. Incidence and location of the TCGA and COSMIC somatic OPCML mutations mapped onto the structure of OPCML. The somatic OPCML mutations identified from the TCGA and COSMIC databases are mapped on to the secondary structure of OPCML. Shown as a bar chart are the amino acid position (X-axis) and incidence (Y-axis), corresponding to mutations identified in domains 1-3. Domain 1 (salmon, residues 42-134) domain 2 (blue, residues 135-222) and domain 3 (green residues 223-310) are shown. For simplicity, the N-terminal secretion signal and the C-terminal GPI-anchor site are not shown.

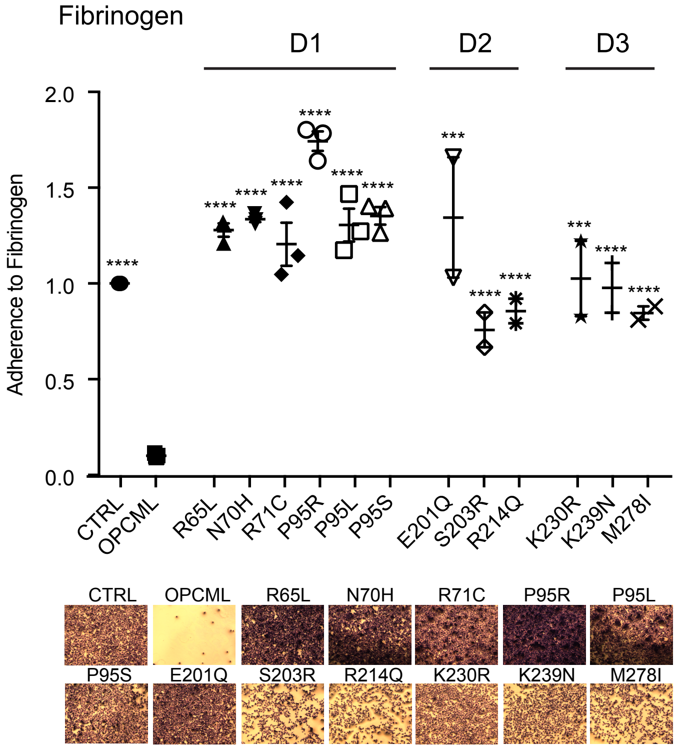

Supplementary Figure 11. D1, D2 and D3 are required to inhibit binding to fibrinogen. SKOV3 cells transduced with an empty vector (CTRL), wild-type (OPCML) or mutant (R65L, N70H, R71C, P95R, P95L, P95S, E201Q, S203R, R214Q, K230R, K239N, M278I) OPCML were plated onto fibrinogen and adhesion tested after 1h. Values have been normalized to CTRL. The images have been taken with a 2.5x objective. The graph shows the mean ± s.e.m of 3 independent experiments. Student t-test compares CTRL and mutants to wild-type OPCML: *** p<0.001, **** p<0.0001.

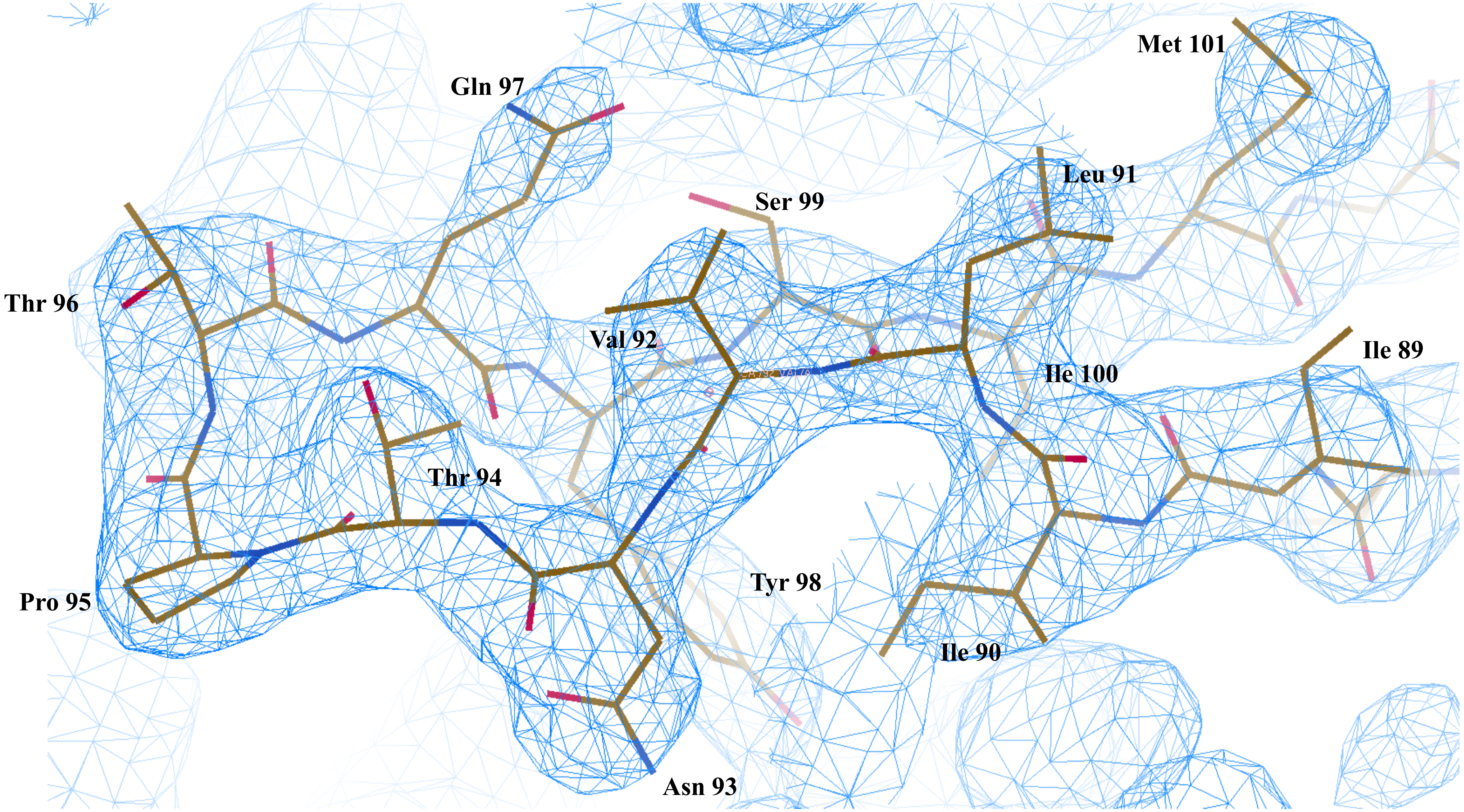

Supplementary Figure 12. Omit map electron density. Omit map showing electron density of the D and E β–strands (residues 89-101) which were omitted from the map calculation. Blue mesh represents |2Fo-Fc|*exp(iαc) electron density, lines represent OPCML model, contoured at 2.0σ.

type of mutations	Domain	AA	Mutation	Tumour	number of patients	Modification / location / comment	mutated AA
1	SP	M1	?	brca	3	Initiator methionine	1
2	SP	G2	A	coadread	1	Signal sequence	2
3	SP	H3	R	lica	1	Signal sequence	3
4			Q	stad	2	Signal sequence	
5	SP	P10	S	coadread	1	Signal sequence	4
6	SP	W11	C	luad	2	Signal sequence	5
7	SP	T14	S	stad	2	Signal sequence	6
8	SP	V17	A	coadread	1	Signal sequence	7
9	SP	S18	Y	ucec	3	Signal sequence	8
10	SP	P26	S	sarc	2	Signal sequence	9
11	SP	R32	C	coadread	1	Signal sequence	10
12			L	blca	3	Signal sequence	
13	SP	A36	D	luad	1	Signal sequence	11
14	1	M42	I	skcm	1	Surface-exposed	12
15	1	D43	H	coadread	2	Surface-exposed	13
16	1	N44	S	paca	1	N-linked glycosylation site	14
17	1	V45	L	lusc	3	Partially surface-exposed	15
18	1	T46	M	uces	2	N-X-S/T site	16
19			M	Endo	1	N-X-S/T site	
20			M	coadread	2	N-X-S/T site	
21	1	G50	E	skcm	2	Surface-exposed	17
22	1	E51	G	thym	2	Surface-exposed	18
23	1	S52	R	stad	2	Surface-exposed	19
24	1	A53	T	cesc	2	Buried	20
25			T	coadread	1	Buried	
26	1	X56	splice	paca	1	Splice site mutation	21

27	1	T58	N	blca	1	Surface-exposed	22
28			K	blca	2	Surface-exposed	
29	1	R62	W	coadread	1	Surface-exposed	23
30			L	lusc	1	Surface-exposed	
31	1	R65	L	luad	1	D1-D1 interface contact residue	24
32	1	N70	H	skcm	1	N-linked glycosylation site	25
33	1	R71	C	coadread	7	D1-D1 interface contact residue	26
34			H	gbm	1	D1-D1 interface contact residue	
35			H	coadread	1	D1-D1 interface contact residue	
36	1	T73	N	ucec	2	D1-D1 interface contact residue	27
37			N	Endo	1	D1-D1 interface contact residue	
38			N	coadread	1	D1-D1 interface contact residue	
39	1	D80	E	skcm	1	D1-D1 interface contact residue	28
40	1	I84	V	stad	3	D1-D1 interface contact residue	29
41			M	lgg-gbm	1	D1-D1 interface contact residue	
42	1	D85	N	skcm	1	D1-D1 interface contact residue	30
43	1	R87	H	coadread	3	D1-D1 interface contact residue	31
44			S	esca	1	D1-D1 interface contact residue	
45	1	T94	S	gbm	3	Surface-exposed	32
46	1	P95	R	Ovary	1	Surface-exposed	33
47			L	Kirc	3	Surface-exposed	
48			S	blca	4	Surface-exposed	
49	1	T96	N	ov	1	Surface-exposed	34
50	1	Q97	H	luad, lusc	4	Partially surface-exposed	35
51	1	Y98	C	Stad	3	Buried	36
52	1	V105	A	Stad	3	Buried	37
53	1	D106	N	skcm	2	Partially surface-exposed	38
54	1	D109	A	coadread	1	D1-D1 interface contact residue	39

55	1	E110	D	coadread	1	Salt bridge between R162 of D2	40
56			K	coadread	3	Salt bridge between R162 of D2	
57			K	skcm	2	Salt bridge between R162 of D2	
58	1	P112	L	skcm	5	Partially surface-exposed	41
59	1	T114	A	ucs	1	D1-D1 interface contact residue	42
60			S	hnsc	2	D1-D1 interface contact residue	
61	1	V117	A	Blca	4	Buried	43
62	1	Q118	E	luad	1	D1-D1 interface contact residue	44
63			E	NCI-60	1	D1-D1 interface contact residue	
64	1	T119	A	coadread	1	Buried	45
65	1	N121	Y	lica	2	Surface-exposed	46
66	1	H122	Y	blca	4	Surface-exposed	47
67	1	P123	S	skcm	2	D1-D1 interface contact residue	48
68	1	T125	M	coadread	2	D1-D1 interface contact residue	49
69	1	R127	W	hnsc	2	D1-D1 interface contact residue	50
70	1	H129	Tfs*2	lica	1	Surface-exposed	51
71	2	D144	E	coadread	2	Surface-exposed	52
72	2	E149	K	skcm	2	D1-D2 interface contact residue	53
73	2	G150	V	cesc	2	Surface-exposed	54
74			E	skcm	2	Surface-exposed	
75			R	skcm	1	Surface-exposed	
76	2	V153	M	coadread	1	Buried	55
77	2	T154	I	coadread	1	Surface-exposed	56
78			S	coadread	1	Surface-exposed	
79	2	T168	A	lica	1	Partially surface-exposed	57
80	2	R170	K	lusc	3	Partially surface-exposed	58
81	2	K175	N	ov	1	Missing in electron density	59
82			N	coadread	1	Missing in electron density	

83	2	G177	V	Stad	2	Missing in electron density	60
84	2	D184	N	Stad	2	Partially surface-exposed	61
85	2	R194	Q	ucec	2	Partially surface-exposed	62
86			Q	Endo	1	Partially surface-exposed	
87	2	D195	G	lica	1	Surface-exposed	63
88	2	E201	K	coadread	3	Partially surface-exposed	64
89			K	skcm	3	Partially surface-exposed	
90			Q	lusc	3	Partially surface-exposed	
91	2	S203	R	luad	4	Partially surface-exposed	65
92			R	hnsc	2	Partially surface-exposed	
93	2	A204	V	Stad	3	Buried	66
94			V	esca	2	Buried	
95	2	N206	K	coadread	1	Buried	67
96	2	D207	E	lusc	3	Surface-exposed	68
97			N	ucec	1	Surface-exposed	
98	2	A209	S	luad	1	Surface-exposed	69
99	2	A210	V	Stad	3	Surface-exposed	70
100			V	prad	1	Surface-exposed	
101	2	P211	L	Pancreas	1	Partially surface-exposed	71
102	2	D212	N	sarc	1	Partially surface-exposed	72
103	2	R214	Q	blca	3	Partially surface-exposed	73
104			W	luad	1	Partially surface-exposed	
105			P	luad	2	Partially surface-exposed	
106	2	V216	*	brca	2	Premature stop codon	74
107	2	V220	E	coadread	1	Buried	75
108	3	P223	S	blca	3	D2-D3 interface contact residue	76
109			S	paca	1	D2-D3 interface contact residue	
110	3	P224	L	lica	1	D2-D3 interface contact residue	77

111			L	skcm	1	D2-D3 interface contact residue	
112	3	Y225	S	Meninges	1	D2-D3 interface contact residue	78
113	3	A229	V	skcm	1	Partially surface-exposed	79
114	3	K230	R	esca	2	Surface-exposed	80
115	3	N231	D	coadread	1	Partially surface-exposed	81
116	3	T232	I	luad	2	Partially surface-exposed	82
117	3	S235	L	skcm	2	Surface-exposed	83
118	3	Q238	K	paca	1	Surface-exposed	84
119	3	K239	N	skcm	1	Surface-exposed	85
120			T	skcm	2	Surface-exposed	
121	3	C244	R	coadread	1	Disulphide with cys 296	86
122	3	A246	G	skcm	1	Buried	87
123	3	P250	S	skcm	2	Surface-exposed	88
124	3	M251	V	Stad	3	Partially surface-exposed	89
125	3	F254	S	luad	1	Buried	90
126	3	Q255	H	lica	2	Partially surface-exposed	91
127			L	luad	2	Partially surface-exposed	
128	3	E259	K	skcm	1	Surface-exposed	92
129	3	E260	*	sarc	1	Premature stop codon	93
130	3	T261	I	coadread	1	Surface-exposed	94
131	3	A264	V	gbm	1	Surface-exposed	95
132	3	G266	C	coadread	2	Surface-exposed	96
133	3	G269	E	hnsc	2	Partially surface-exposed	97
134	3	R271	M	coadread	2	Surface-exposed	98
135			K	Blca	3	Surface-exposed	
136	3	I272	N	coadread	1	Buried	99
137	3	E273	K	skcm	1	Partially surface-exposed	100
138	3	R277	H	lica	1	Partially surface-exposed	101

139			H	ucs	1	Partially surface-exposed	
140			H	NCI-60	1	Partially surface-exposed	
141			P	luad	1	Partially surface-exposed	
142			C	ucs	1	Partially surface-exposed	
143	3	M278	I	ov	3	Partially surface-exposed	102
144	3	T282	N	paca	1	Buried	103
145	3	F284	V	esca	1	Partially surface-exposed	104
146	3	D290	V	luad	1	Buried	105
147	3	G292	V	lica	2	Surface-exposed	106
148	3	N300	K	coadread	3	Buried	107
149	3	G303	E	coadread	1	Surface-exposed	108
150	3	N306	K	prad	2	N-linked glycosylation site	109
151	C-term	P314	S	skcm	2	C-terminal GPI anchor site	110
152	C-term	N322	I	blca	3	C-terminal GPI anchor site	111
153	C-term	S323	L	skcm	1	C-terminal GPI anchor site	112
154	C-term	L328	Kfs*6	paca	1	C-terminal GPI anchor site	113
155	C-term	L331	I	coadread	3	C-terminal GPI anchor site	114
156			H	ov	1	C-terminal GPI anchor site	
157			F	skcm	1	C-terminal GPI anchor site	
158	C-term	W332	C	hnsc	2	C-terminal GPI anchor site	115
159	C-term	A339	V	skcm	2	C-terminal GPI anchor site	116
160	C-term	F341	L	Stad	1	C-terminal GPI anchor site	117
161	C-term	*346	Sext*1	coadread	2	C-terminal GPI anchor site	118
162	C-term	*346	Rext*1	Stad	2	C-terminal GPI anchor site	119

Supplementary Table 1. Mutations identified in cancer patients. The type of mutation, the location in the domains, the cancer of origin and the possible effect on the 3D structure are listed as indicated.

Interface	Interface area (Å²)	Solvation free energy gain (ΔiG)	Symmetry operator	Chain	Residue number/atom	Distance (Å)	Chain	Residue number/atom
1	873.9	-9.5	x,y,z	Hydrogen bonds				
				B	THR 114[OG1]	3.78	A	SER 72[OG]
				B	ARG 65[NH2]	2.96	A	ASP 80[OD1]
				B	SER 72[OG]	3.72	A	THR 114[OG1]
				B	ILE 74[N]	3.76	A	SER 116[OG]
				B	SER 72[OG]	2.98	A	ARG 127[NH2]
				B	ASP 80[OD1]	2.68	A	ARG 65[NH2]
				B	SER 116[OG]	3.87	A	ILE 74[N]
				Salt bridges				
				B	ARG 65[NE]	3.77	A	ASP 80[OD1]
				B	ARG 65[NH2]	2.96	A	ASP 80[OD1]
				B	ARG 65[NH1]	3.02	A	ASP 80[OD2]
				B	ARG 65[NE]	3.61	A	ASP 80[OD2]
				B	ARG 65[NH2]	3.09	A	ASP 80[OD2]
				B	ASP 80[OD1]	3.61	A	ARG 65[NE]
				B	ASP 80[OD1]	2.68	A	ARG 65[NH2]
				B	ASP 80[OD2]	3.70	A	ARG 65[NE]
				B	ASP 80[OD2]	2.92	A	ARG 65[NH2]
				B	ASP 80[OD2]	2.94	A	ARG 65[NH1]
2	553	-6.8	y-1,x,-z+2	Hydrogen bonds				
				B	SER 235[OG]	2.96	A	ASN 44[O]
				B	ALA 316[N]	2.91	A	ARG 56[O]
				B	ALA 317[N]	3.07	A	SER 126[OG]
				B	LEU 318[N]	3.17	A	THR 58[O]
				B	SER 235[OG]	3.17	A	THR 46[N]
				B	GLN 238[OE1]	3.68	A	GLN 133[NE2]
				B	ALA 316[O]	2.89	A	THR 58[N]
				B	LEU 318[O]	3.22	A	ASP 60[N]
3	511.2	-8.6	y,x-1,-z+2	Hydrogen bonds				
				B	ASN 44[ND2]	3.65	A	SER 235[OG]
				B	MET 42[N]	3.79	A	GLY 315[O]
				B	THR 58[N]	3.06	A	ALA 316[O]
				B	ASP 60[N]	3.57	A	LEU 318[O]
				B	ARG 56[O]	3.05	A	ALA 316[N]
				B	SER 126[OG]	3.04	A	ALA 317[N]
				B	THR 58[O]	3.12	A	LEU 318[N]

4	394.3	-0.8	-<br>y+1/2,x+1/2,z+1/4	Hydrogen bonds				
				B	ASN 93[N]	2.93	B	ILE 226[O]
				B	ARG 56[NH1]	3.47	B	ASN 306[O]
				B	ARG 56[NH2]	3.42	B	ASN 306[O]
				B	ARG 56[NH2]	3.05	B	ASN 306[OD1]
				B	LEU 91[O]	2.89	B	THR 305[OG1]
				B	LEU 91[O]	2.89	B	ILE 226[N]
5	368.7	-2	-y+1/2,x-1/2,z+1/4	Hydrogen bonds				
				A	ARG 56[NH1]	3.52	A	ASN 306[O]
				A	ARG 56[NH2]	3.32	A	ASN 306[O]
				A	ARG 56[NH2]	3.01	A	ASN 306[OD1]
				A	ASN 93[N]	2.87	A	ILE 226[O]
				A	LEU 91[O]	2.81	A	ILE 226[N]
				A	LEU 91[O]	2.88	A	THR 305[OG1]
6	235.7	-1.6		Hydrogen bonds				
				B	LYS 258[NZ]	2.87	A	GLU 259[OE1]
				Salt bridges				
				B	LYS 258[NZ]	2.87	A	GLU 259[OE1]

Supplementary Table 2. Shown are the 6 largest contact areas as seen in the OPCML crystal structure and the buried surface area along with the contact residues and distances involved are listed, within 4 Å. Figures were calculated using PISA. The largest contact surface corresponds to the D1-D1 homodimerization interface and contains 874 Å^2 of interface area. The next 5 largest sites comprise consecutively less surface area and fewer amino acid contact sites.
