# Supplementary material for: Inactivating mutations and X-ray crystal structure of the tumor suppressor OPCML reveal cancer-associated functions
Source: Nat Commun. 2019 Jul 17;10:3134. doi: 10.1038/s41467-019-10966-8 (PMC6637204; doi:10.1038/s41467-019-10966-8)
Supplement: Supplementary file 3 — Reporting Summary [file 41467_2019_10966_MOESM3_ESM.pdf]

nature research

Corresponding author(s): Hani Gabra, Lawrence Stern, Chiara Recchi

Last updated by author(s): Apr 15, 2019

Reporting Summary

Nature Research wishes to improve the reproducibility of the work that we publish. This form provides structure for consistency and transparency in reporting. For further information on Nature Research policies, see Authors & Referees and the Editorial Policy Checklist.

Statistics

For all statistical analyses, confirm that the following items are present in the figure legend, table legend, main text, or Methods section.

n/a	Confirmed	
☐	☒	The exact sample size (*n*) for each experimental group/condition, given as a discrete number and unit of measurement
☐	☒	A statement on whether measurements were taken from distinct samples or whether the same sample was measured repeatedly
☐	☒	The statistical test(s) used AND whether they are one- or two-sided<br>*Only common tests should be described solely by name; describe more complex techniques in the Methods section.*
☐	☒	A description of all covariates tested
☐	☒	A description of any assumptions or corrections, such as tests of normality and adjustment for multiple comparisons
☐	☒	A full description of the statistical parameters including central tendency (e.g. means) or other basic estimates (e.g. regression coefficient) AND variation (e.g. standard deviation) or associated estimates of uncertainty (e.g. confidence intervals)
☒	☐	For null hypothesis testing, the test statistic (e.g. *F*, *t*, *r*) with confidence intervals, effect sizes, degrees of freedom and *P* value noted<br>*Give P values as exact values whenever suitable.*
☒	☐	For Bayesian analysis, information on the choice of priors and Markov chain Monte Carlo settings
☒	☐	For hierarchical and complex designs, identification of the appropriate level for tests and full reporting of outcomes
☒	☐	Estimates of effect sizes (e.g. Cohen's *d*, Pearson's *r*), indicating how they were calculated

Our web collection on statistics for biologists contains articles on many of the points above.

Software and code

Policy information about availability of computer code

Data collection	no software used
Data analysis	no software used

For manuscripts utilizing custom algorithms or software that are central to the research but not yet described in published literature, software must be made available to editors/reviewers. We strongly encourage code deposition in a community repository (e.g. GitHub). See the Nature Research guidelines for submitting code & software for further information.

Data

Policy information about availability of data

All manuscripts must include a data availability statement. This statement should provide the following information, where applicable:

- Accession codes, unique identifiers, or web links for publicly available datasets
- A list of figures that have associated raw data
- A description of any restrictions on data availability

All data generated or analyzed during this study are included in this published article
(and in its accompanying Supplementary Information).
The atomic coordinates and structure factors for the crystal structure of the OPCML protein determined in this study have
been deposited in the Protein Data Bank with accession code 5UV6.

Field-specific reporting

Please select the one below that is the best fit for your research. If you are not sure, read the appropriate sections before making your selection.

☒ Life sciences ☐ Behavioural & social sciences ☐ Ecological, evolutionary & environmental sciences

For a reference copy of the document with all sections, see nature.com/documents/nr-reporting-summary-flat.pdf

Life sciences study design

All studies must disclose on these points even when the disclosure is negative.

Sample size	Sufficient replicates for statistical significance, at least three and often more were undertaken
Data exclusions	No data exclusions were necessary in this study
Replication	Data successfully replicated in independent experiments and can be reproduced
Randomization	Not applicable
Blinding	Not applicable

Reporting for specific materials, systems and methods

We require information from authors about some types of materials, experimental systems and methods used in many studies. Here, indicate whether each material, system or method listed is relevant to your study. If you are not sure if a list item applies to your research, read the appropriate section before selecting a response.

Materials & experimental systems

n/a	Involved in the study	
☐	☒	Antibodies
☐	☒	Eukaryotic cell lines
☒	☐	Palaeontology
☐	☒	Animals and other organisms
☒	☐	Human research participants
☒	☐	Clinical data

Methods

n/a	Involved in the study	
☒	☐	ChIP-seq
☒	☐	Flow cytometry
☒	☐	MRI-based neuroimaging

Antibodies

Antibodies used	anti OPCML (goat pAb, #AF2777, R&D Systems),<br>phospho-AKT (Thr308 D25E6 XP® rabbit mAb, #13038, Cell Signaling),<br>AKT (pan 11E7 rabbit mAb, #4685 Cell Signaling),<br>phospho-ERK1 (pT202/pY204) + phospho-ERK2 (pT185/pY187) (mouse mAb, ab50011, Abcam),<br>ERK1/2 (rabbit pAb, ab17942, Abcam) and anti-GAPDH (Cell Signalling)
Validation	validation was according to manufacturers instructions on their websites - see above

Eukaryotic cell lines

Policy information about cell lines

Cell line source(s)	SKOV3, HEK293T and HCT116 cells were purchased from ATCC. PEA1 and PEO1 cells were acquired from Cancer Research UK.<br>All the cell lines were purchased by ATCC or CRUK as stated in the manuscript.
Authentication	Cell lines were not further authenticated
Mycoplasma contamination	Mycoplasma testing was regularly conducted to assure that all cells used were mycoplasma free at all times
Commonly misidentified lines<br>(See ICLAC register)	No commonly misidentified lines were used in this study

Animals and other organisms

Policy information about studies involving animals; ARRIVE guidelines recommended for reporting animal research

Laboratory animals	Eight-week old athymic nude female mice and chick chorioallantoic model Fertilised chicken eggs
Wild animals	study did not involve wild animals
Field-collected samples	study did not involve samples collected from the field
Ethics oversight	Home Office project licence reference: PPL 70/7997 and by the Imperial College's Animal Welfare and Ethical Review Body

Note that full information on the approval of the study protocol must also be provided in the manuscript.
