# Supplementary material for: Inactivating mutations and X-ray crystal structure of the tumor suppressor OPCML reveal cancer-associated functions
Source: Nat Commun. 2019 Jul 17;10:3134. doi: 10.1038/s41467-019-10966-8 (PMC6637204; doi:10.1038/s41467-019-10966-8)
Supplement: Supplementary file 4 — Source Data [file 41467_2019_10966_MOESM4_ESM.zip › Fig 4/Fig 4E-F-G.pptx]

## Slide 1
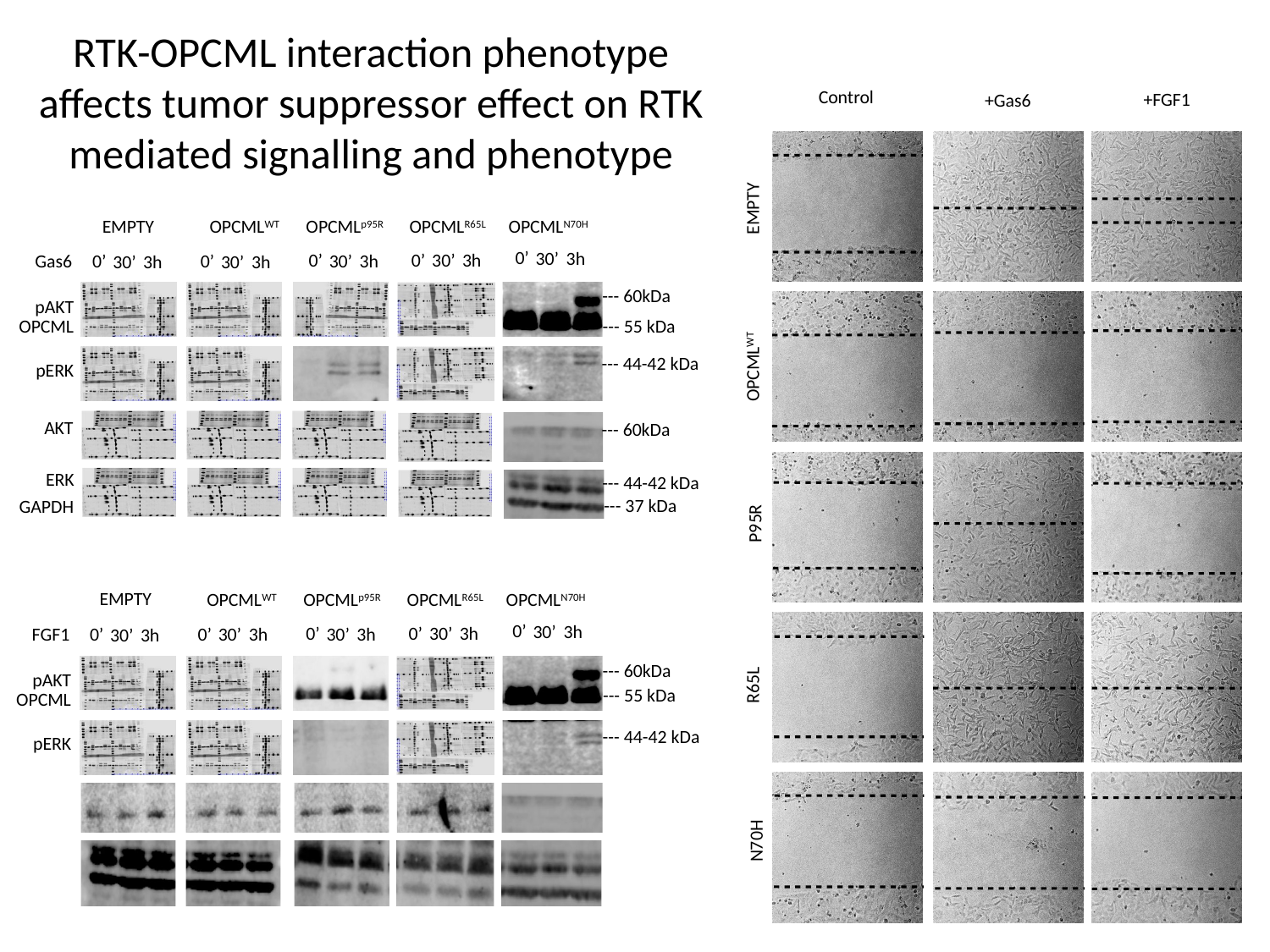

RTK-OPCML interaction phenotype affects tumor suppressor effect on RTK mediated signalling and phenotype
Control
+FGF1
+Gas6
EMPTY
EMPTY
OPCMLWT
OPCMLp95R
OPCMLR65L
OPCMLN70H
0’
30’
3h
0’
30’
3h
0’
30’
3h
0’
30’
3h
Gas6
0’
30’
3h
--- 60kDa
pAKT
--- 55 kDa
OPCML
--- 44-42 kDa
pERK
--- 37 kDa
GAPDH
OPCMLWT
AKT
--- 60kDa
ERK
--- 44-42 kDa
P95R
EMPTY
OPCMLWT
OPCMLp95R
OPCMLR65L
OPCMLN70H
0’
30’
3h
0’
30’
3h
0’
30’
3h
0’
30’
3h
FGF1
0’
30’
3h
--- 60kDa
pAKT
--- 55 kDa
OPCML
pERK
R65L
--- 44-42 kDa
N70H
