# Supplementary figures and images for: Inactivating mutations and X-ray crystal structure of the tumor suppressor OPCML reveal cancer-associated functions
Source: Nat Commun. 2019 Jul 17;10:3134. doi: 10.1038/s41467-019-10966-8 (PMC6637204; doi:10.1038/s41467-019-10966-8)

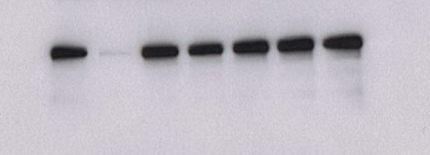

Supplement: Supplementary file 4 — Source Data [file 41467_2019_10966_MOESM4_ESM.zip › Fig 3/Fig 3G_Her2.jpg]

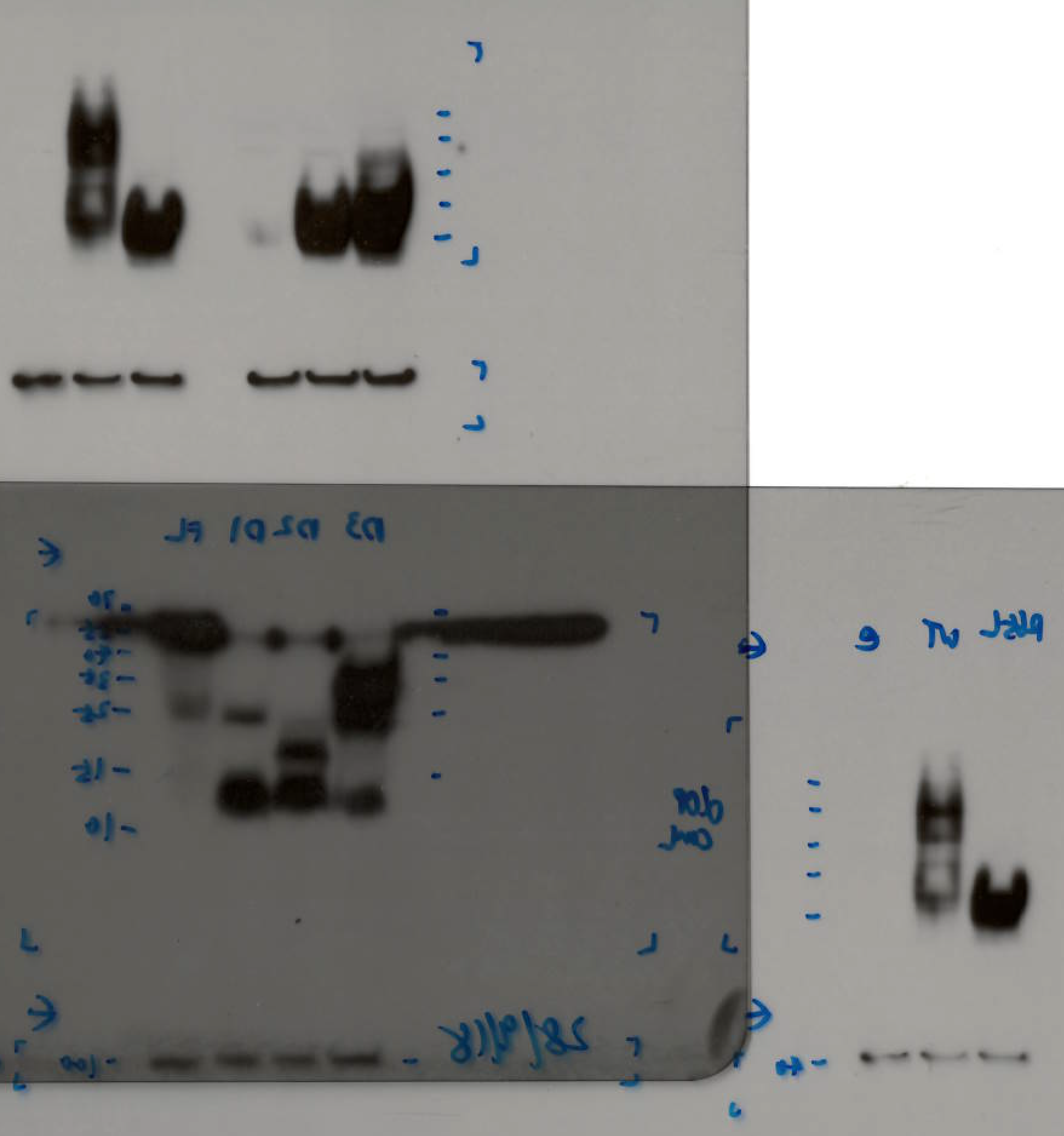

Supplement: Supplementary file 4 — Source Data [file 41467_2019_10966_MOESM4_ESM.zip › Fig 3/Fig 3A_dimer.tif]

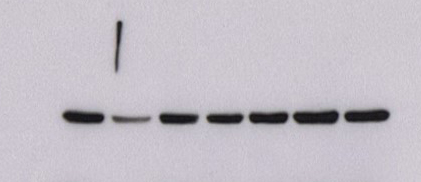

Supplement: Supplementary file 4 — Source Data [file 41467_2019_10966_MOESM4_ESM.zip › Fig 3/Fig 3G_pAKT.jpg]

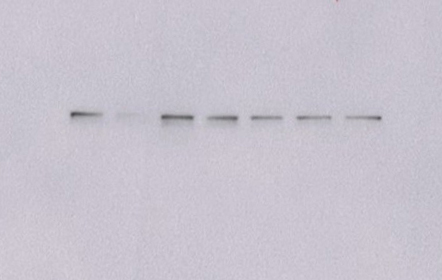

Supplement: Supplementary file 4 — Source Data [file 41467_2019_10966_MOESM4_ESM.zip › Fig 3/Fig 3G_pHER2.jpg]

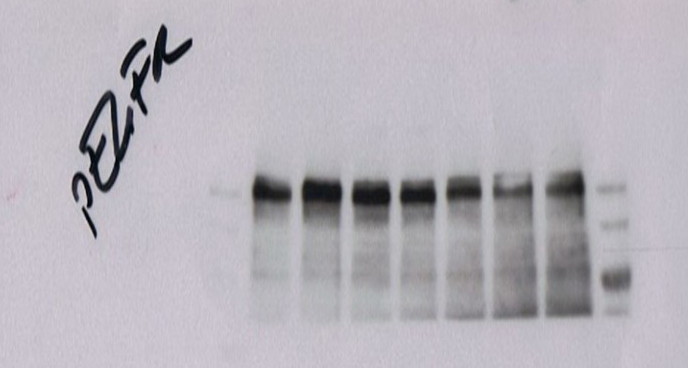

Supplement: Supplementary file 4 — Source Data [file 41467_2019_10966_MOESM4_ESM.zip › Fig 3/Fig 3G_pEGFR.jpg]

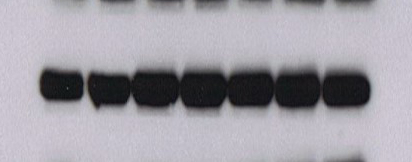

Supplement: Supplementary file 4 — Source Data [file 41467_2019_10966_MOESM4_ESM.zip › Fig 3/Fig 3G_AKT.jpg]

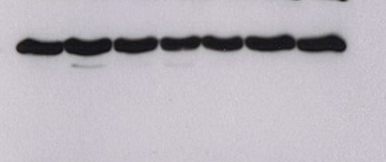

Supplement: Supplementary file 4 — Source Data [file 41467_2019_10966_MOESM4_ESM.zip › Fig 3/Fig 3G_GAPDH.jpg]

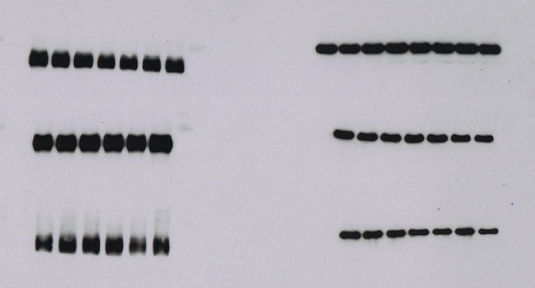

Supplement: Supplementary file 4 — Source Data [file 41467_2019_10966_MOESM4_ESM.zip › Fig 3/Fig 3A_western OPCML_GAPDH.tif]

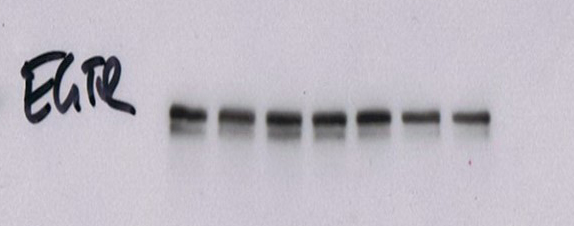

Supplement: Supplementary file 4 — Source Data [file 41467_2019_10966_MOESM4_ESM.zip › Fig 3/Fig 3G_EGFR.jpg]

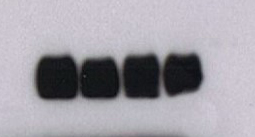

Supplement: Supplementary file 4 — Source Data [file 41467_2019_10966_MOESM4_ESM.zip › Fig 5/Fig 5B_Lysates OPCML.jpg]

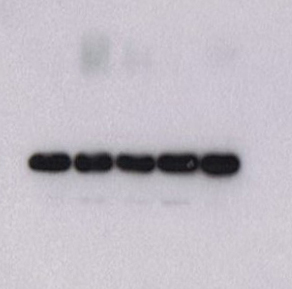

Supplement: Supplementary file 4 — Source Data [file 41467_2019_10966_MOESM4_ESM.zip › Fig 5/Fig 5B_Lysates GAPDH.jpg]

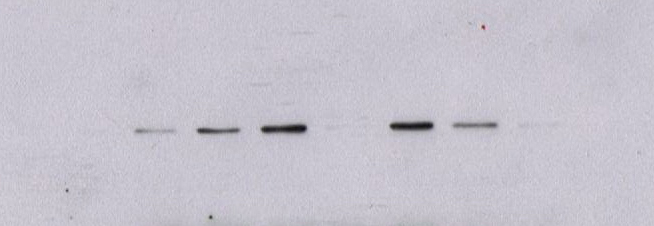

Supplement: Supplementary file 4 — Source Data [file 41467_2019_10966_MOESM4_ESM.zip › Fig 5/Fig 5D_CO pAKT_Image (2)*.jpg]

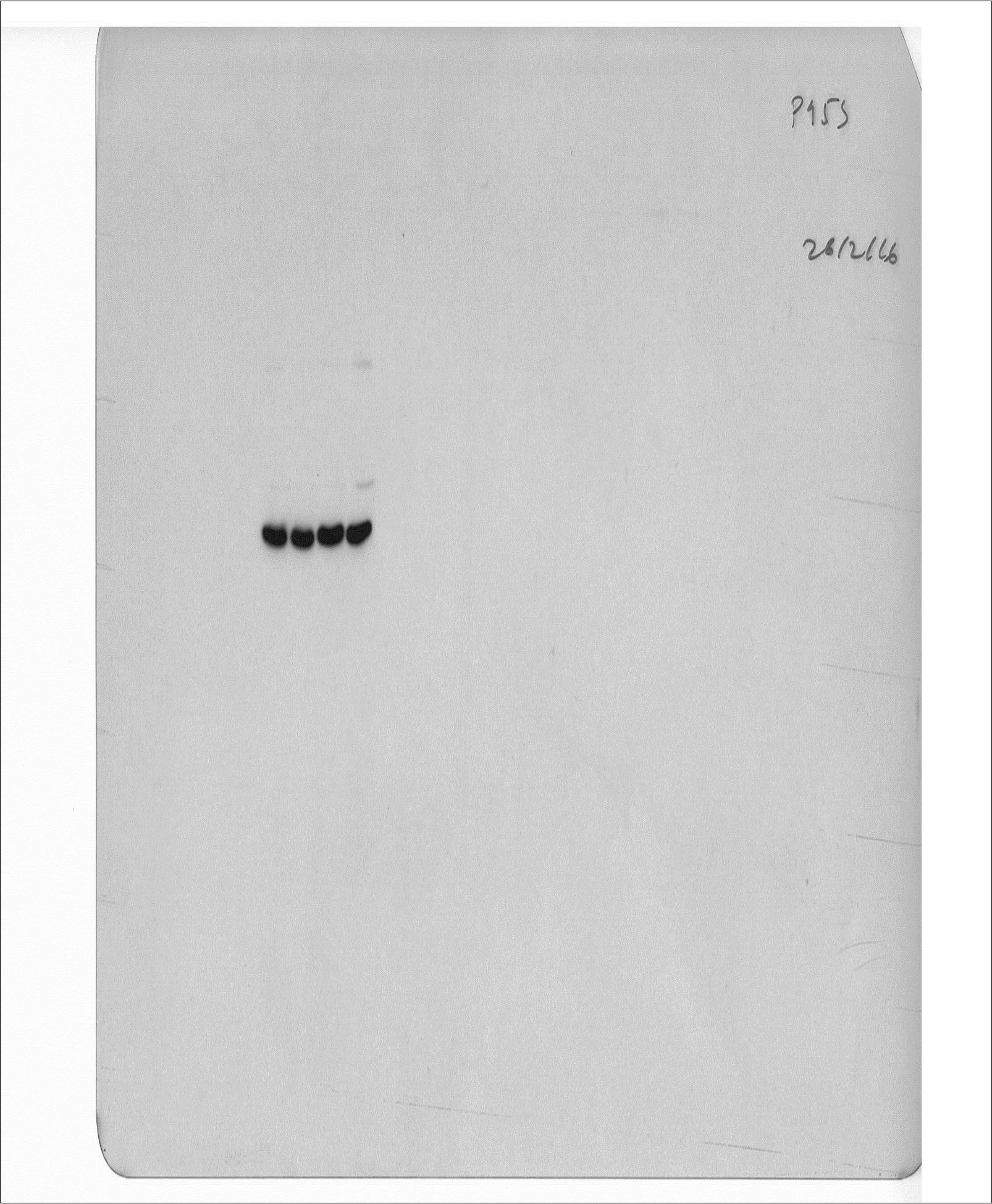

Supplement: Supplementary file 4 — Source Data [file 41467_2019_10966_MOESM4_ESM.zip › Fig 5/Fig 5D_S GAPDH_Image (16)*.jpg]

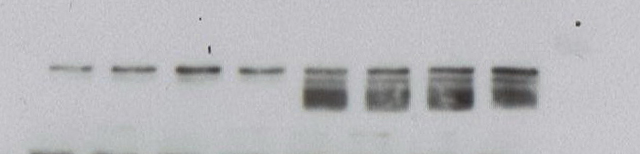

Supplement: Supplementary file 4 — Source Data [file 41467_2019_10966_MOESM4_ESM.zip › Fig 5/Fig 5D_CO tAKT_Image (22)*.jpg]

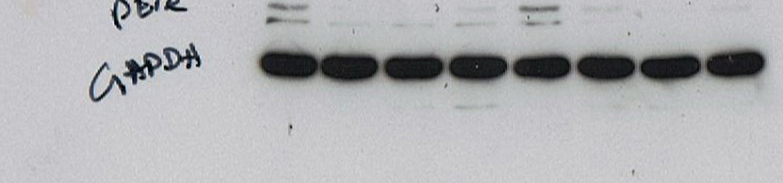

Supplement: Supplementary file 4 — Source Data [file 41467_2019_10966_MOESM4_ESM.zip › Fig 5/Fig 5D_RL GAPDH_Image (82)*.jpg]

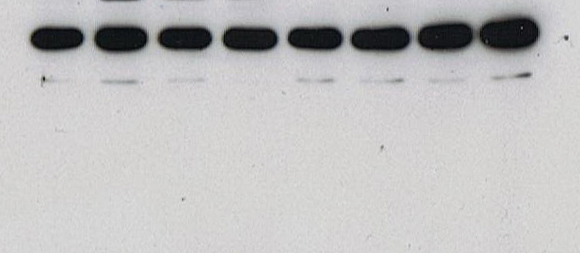

Supplement: Supplementary file 4 — Source Data [file 41467_2019_10966_MOESM4_ESM.zip › Fig 5/Fig 5D_CO GAPDH_Image (21)*.jpg]

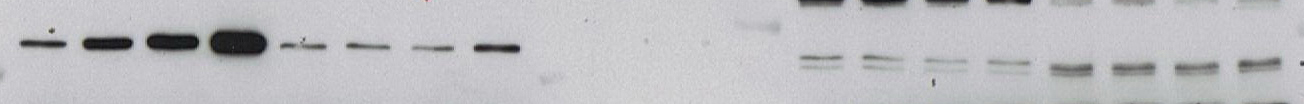

Supplement: Supplementary file 4 — Source Data [file 41467_2019_10966_MOESM4_ESM.zip › Fig 5/Fig 5D_RL ptAKT_Image (12)*.jpg]

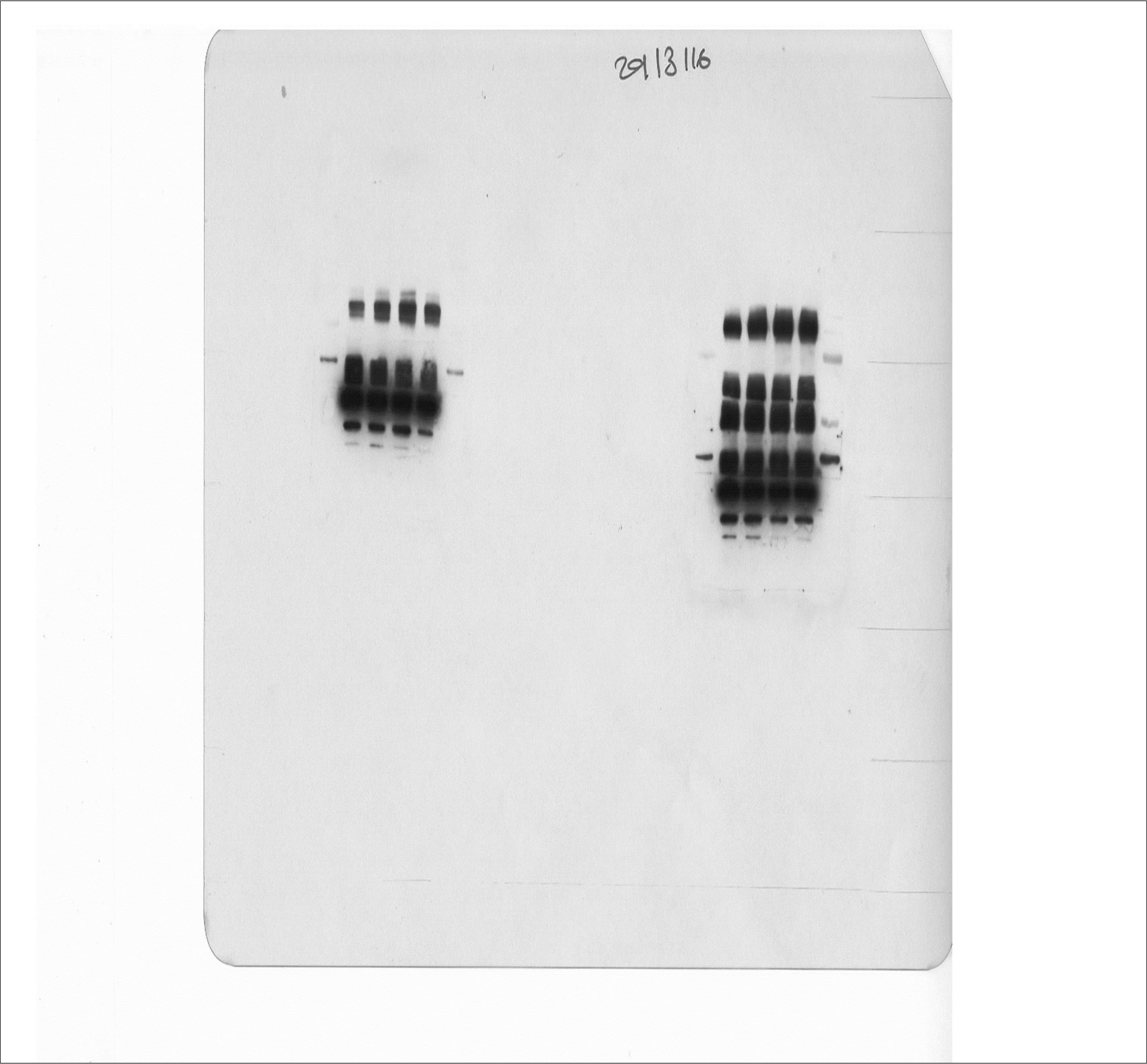

Supplement: Supplementary file 4 — Source Data [file 41467_2019_10966_MOESM4_ESM.zip › Fig 5/Fig 5D_S ptAKT_Image (13)*.jpg]

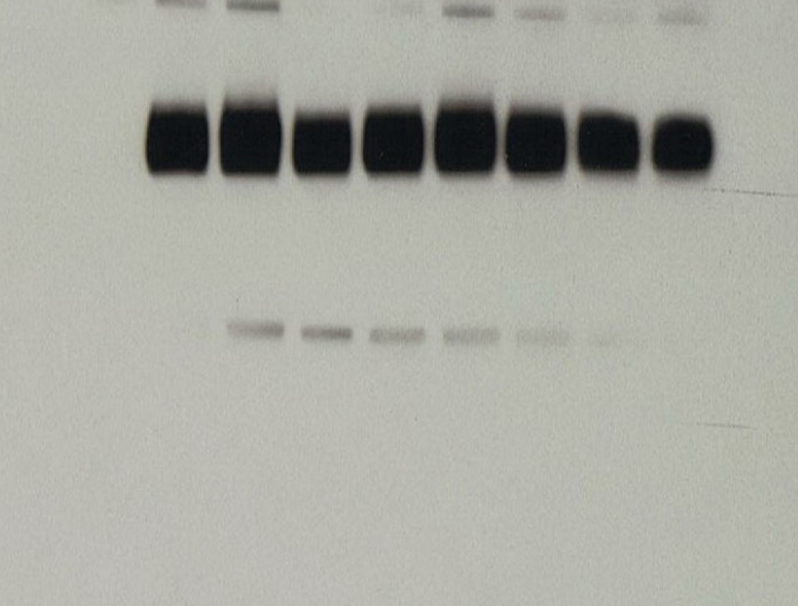

Supplement: Supplementary file 4 — Source Data [file 41467_2019_10966_MOESM4_ESM.zip › Fig 6/Fig 6A_western OPCML.jpg]

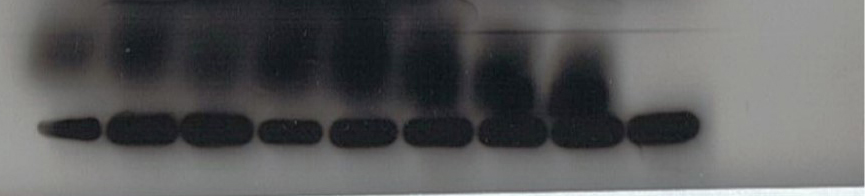

Supplement: Supplementary file 4 — Source Data [file 41467_2019_10966_MOESM4_ESM.zip › Fig 6/Fig 6A_western GAPDH.jpg]
